# An analysis of the dual burden of childhood stunting and wasting in Myanmar: a copula geoadditive modelling approach

**DOI:** 10.1017/S1368980024000193

**Published:** 2024-01-19

**Authors:** Dhiman Bhadra

**Affiliations:** Operations and Decision Sciences Area, Indian Institute of Management Ahmedabad, Ahmedabad, Gujarat 380015, India

**Keywords:** Copula geoadditive modelling, Childhood stunting, Joint probabilities, Myanmar demographic and health surveys

## Abstract

**Objective:**

To analyse the spatial variation and risk factors of the dual burden of childhood stunting and wasting in Myanmar.

**Design:**

Analysis was carried out on nationally representative data obtained from the Myanmar Demographic and Health Survey conducted during 2015–2016. Childhood stunting and wasting are used as proxies of chronic and acute childhood undernutrition. A child with standardised height-for-age Z score (HAZ) below –2 is categorised as stunted while that with a weight-for-height Z score (WHZ) below –2 as wasted.

**Setting:**

A nationally representative sample of households from the fifteen states and regions of Myanmar.

**Participants:**

Children under the age of five ( 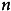 4162).

**Results:**

Overall marginal prevalence of childhood stunting and wasting was 28·9 % (95 % CI 27·5, 30·2) and 7·3 % (95 % CI 6·5, 8·0) while their concurrent prevalence was 1·6 % (95 % CI 1·2, 2·0). The study revealed mild positive association between stunting and wasting across Myanmar. Both stunting and wasting had significant spatial variation across the country with eastern regions having higher burden of stunting while southern regions having higher prevalence of wasting. Child age and maternal WHZ score had significant non-linear association with both stunting and wasting while child gender, ethnicity and household wealth quintile had significant association with stunting.

**Conclusion:**

The study provides data-driven evidence about the association between stunting and wasting and their spatial variation across Myanmar. The resulting insights can aid in the formulation and implementation of targeted, region-specific interventions towards improving the state of childhood undernutrition in Myanmar.

Childhood undernutrition is a major contributor of childhood mortality worldwide. It is estimated that nearly 50 % of deaths in children under 5 is due to some form of undernutrition^(1)^ while majority of these deaths occur in low- and middle-income countries. Undernutrition can be categorised into four broad classes, namely stunting or low height-for-age, wasting or low weight-for-height, underweight or low weight-for-age and lastly mineral and vitamin deficiency. It is a subset of childhood malnutrition which incorporate measures of overnutrition such as childhood overweight and obesity as well. As per 2020 estimates, 149·2 million children under 5 are stunted, 45 million are wasted and 462 million are underweight^(2)^. These correspond to 22 %, 6·7 % and 68·18 % of all children under 5 globally. Although childhood stunting has steadily decreased since 2000, childhood wasting has not shown any significant decline and persists at alarming rates in South Asia and sub-Saharan Africa.

Undernutrition is primarily attributed to lack of access to proper nutrition during the first 1000 days from conception. It makes a child more susceptible to common infections, delays recovery and increases the risk of mortality. Undernutrition during childhood has both short- and long-term consequences on various aspects of health and well-being. For instance, it is associated with increased risk of mortality and morbidity during childhood^(3,4)^ and reduced neurocognitive development and elevated risk of non-communicable diseases during adulthood^(5)^. It has long-term consequences on physical and psychological well-being, educational attainment and labour market productivity of an individual^(6,7)^ and can potentially hinder a country’s human, social and economic progress^(8–10)^. Overall, early childhood undernutrition leads to an intergenerational cycle of undernutrition and prevents large section of the population to escape poverty traps through a vicious cycle of deprivation. Increased awareness of the debilitating consequences of childhood undernutrition has led to its being identified as a major global health priority by international organisations such as United Nations and WHO. In doing so, concrete timelines have been set for meeting global nutrition targets such as sustainable development goals, specifically SDG 2, that call for an end to all possible forms of hunger and malnutrition by 2030.

There exists considerable literature on the determinants of childhood undernutrition specifically as it manifests through stunting and wasting^(11–13)^. These can be at the individual, maternal and household level such as child’s age, gender, mother’s health, education and working status as well as household location and wealth status^(14)^. In addition, dietary diversity, complimentary feeding practices as well as access to potable water and sanitation have been shown to have significant influence on childhood undernutrition^(15)^. Recent studies have also explored the impact of climatic and environmental anomalies in precipitating childhood undernutrition^(16,17)^.

Existing studies on childhood undernutrition usually model the covariate–response relationship for stunting and wasting separately. However, it is entirely conceivable that childhood stunting and wasting are related in a particular population and can vary across regions. In studies carried out across Africa, Americas, Asia and Eastern Mediterranean, it has been found that the prevalence of stunting and wasting as well as their association varies considerably across regions^(18)^. Specifically, it is observed that stunting and wasting have low correlations in Africa and Latin America but high positive association in Asia and the Eastern Mediterranean regions. In a recent review study on low- and middle-income countries, stunting and wasting were found to have a strong association whereby episodes of wasting contribute to stunting while stunting leads to wasting as well, albeit to a lesser extent^(19)^. Moreover, children with concurrent stunting and wasting were found to have the highest risk of mortality compared to children who were either stunted or wasted. In this study, we explore the spatial variation and antecedents of childhood stunting and wasting in Myanmar while accounting for the possible association between the two growth measures.

Myanmar is the second largest country in Southeast Asia as well as one of the poorest. Since its independence in 1948, Myanmar has been majorly ruled by an oppressive military junta, which was responsible for wide spread ethnic persecution and severe curtailment of democratic and civil liberties of its citizens. This led to a gradual deterioration of the social, industrial and economic health of the country^(20)^. In addition to political and ethnic upheavals, Myanmar is also vulnerable to a wide range of environmental disturbances. Since 2002, it is estimated that more than 13 million people have been affected by natural disasters in Myanmar^(21)^, each of which has resulted in massive displacement of populace and caused widespread damage and destruction of crops, livelihoods and property. Scenarios like the above create a volatile ecosystem that breeds food insecurity and deprivation^(22)^. Childhood undernutrition is a direct consequence of this, since young children are particularly susceptible to nutritional deficiencies when households experience food insecurity and shortages. Needless to say, childhood undernutrition has been a major public health concern in Myanmar with over 1·3 million or nearly 29 % of under five children being stunted and more than 300 000, or nearly 7 % wasted^(21)^. These are some of the highest rates of stunting and wasting among children under five among the Association of Southeast Asian Nations.

Existing research on the determinants of childhood undernutrition for Myanmar is relatively recent but provides crucial pan-country perspectives about the drivers of childhood stunting and wasting^(11,14,23)^ as well as the dual burden of childhood stunting and maternal overweight and obesity^(24)^. However, in these studies, the determinant-response pathways are modelled separately for each of the malnutrition indices. In this context, the current study provides four novel contributions to the literature. First, we jointly model childhood stunting and wasting in relation to various socio-economic and demographic determinants. Second, we use a flexible modelling approach to incorporate non-linear association between the determinants and the undernutrition indices. Third, we account for spatial variations of stunting, wasting as well as their association across the regions of Myanmar by incorporating both within-region and between-region spatial effects. Finally, we quantify the joint likelihood all possible combinations of stunting and wasting across all the regions and produce spatial maps of the same. We implement these through a comprehensive modelling framework based on the copula geoadditive modelling approach^(25,26)^. To our knowledge, this is possibly the first study that implements a joint modelling framework to analyse the dual burden of childhood stunting and wasting based on nationally representative data from any country in general and Myanmar in particular. We hope that the results of this study will inform policy makers and programme managers about regional variation in the joint prevalence of childhood stunting and wasting in Myanmar and will aid in the design and implementation of regionally sensitive nutritional interventions for improving the state of childhood undernutrition in the country.

## Methods

### Myanmar Demographic Health Survey

We used data from the Myanmar Demographic Health Survey (MDHS) carried out during December 2015 to July 2016 for the purpose of our analysis. This is the first and only Demographic Health Survey (DHS) to be carried out in Myanmar till date and was funded by USAID and Three Millennium Development Goals Fund and carried out by the Ministry of Health and Sports. The survey provides information on health and nutritional characteristics of a nationally representative sample of women in their reproductive age (15–49 years).

Myanmar is administratively divided into seven regions, seven states and one union territory. The regions are Ayeyarwady, Bago, Magway, Mandalay, Sagaing, Tanintharyi and Yangon while the states are Kachin, Kayah, Kayin, Chin, Mon, Rakhine and Shan. The national capital namely NayPyidaw constitutes the sole union territory. The MDHS followed a two-stage stratified-cluster sampling scheme to select a nationally representative sample of households from the entire country. In the first stage, each of the above states and regions was divided into rural and urban segments, each of which formed a separate sampling stratum. In the second stage, a random sample of household clusters was selected independently from each of those strata using proportional allocation. This led to a total of 442 clusters, of which 123 were urban and 319 were rural. Lastly, thirty households were selected from each cluster using equal probability systematic sampling, resulting in a representative sample of 13 260 households from the country. Of them, 12 500 households were interviewed. From each of the selected households, information was collected from all women aged 15–49 years who were either permanent residents or who stayed in the households the night before the interview was administered. For the purpose of our analysis, we used the children’s data file, which had information on 4815 children born within 5 years prior to the interview. Of those, 653 children had to be excluded for having missing values on various child, maternal and household-specific attributes as well as for having height-for-age and weight-for-height Z scores (HAZ and WHZ) below –6 or above 6. Upon exclusion, the final analysis-ready dataset consisted of complete observations on all the necessary variables for 4162 children and their mothers. Spatial mapping of childhood stunting and wasting was carried out at the regional level since that is the lowest administrative level at which publicly available spatial data files of Myanmar are available. The MDHS dataset too mapped the sampled children with their state and region of residence.

Ethical approval was not required as the DHS datasets are publicly available and use accepted procedures for data collection with ethically approved guidelines and informed consent from the participants. Details regarding the sample design and survey instruments are provided elsewhere^(27)^.

### Study variables

In this study, childhood stunting and wasting are used as proxies for chronic and acute childhood undernutrition, respectively. Anthropometric measures of children’s HAZ and WHZ scores were used to construct indicators of these indices. These scores and the corresponding thresholds for attribution are based on growth standard median metrics formulated by the WHO for children below 5 years. Specifically, children whose standardised HAZ and WHZ scores are below –2 are labelled as stunted and wasted, respectively. Children with scores more than 6 or less than –6 are treated as outliers and dropped. Thus, the dependent variables are binary with categories ‘stunted’ and ‘not stunted’ and ‘wasted’ and ‘not wasted’, respectively.

We considered the following risk factors of stunting and wasting based on those accepted in existing literature as having some association with these conditions: child gender (1 if male, 2 if female)^(11,24)^, child age (months)^(11)^, maternal age at first birth (in years)^(24)^, maternal working status (1 if working, 0 if not working)^(11,24)^, maternal educational attainment (0 if no education, 1 if primary, 2 if secondary, 3 if higher secondary)^(24)^, household location (1 if rural, 0 if urban)^(24)^, gender of household head (1 if male, 2 if female)^(17)^, household wealth quintile (1 if poorest, 2 if poorer, 3 if middle, 4 if richer, 5 if richest)^(11,24)^, toilet facility (1 if improved, 0 if not improved)^(11,13)^, on-premise drinking water (1 if available, 0 if not available)^(11,13)^ and water treatment (1 if done, 0 if not done). The categorisation for toilet facility and on-premise drinking water was done as per guidelines laid down in the DHS manual. We also considered ethnicity of a child as well as standardised HAZ and WHZ scores of the mother as proxies for maternal stunting and wasting status. As per ethnicity is concerned, a child hailing from Chin, Kachin, Kayah, Kayin, Mon, Rakhine or Shan was considered as ‘minority’ (ethnicity = 0) else a ‘majority’ (ethnicity = 1). This categorisation is as per country demographic distribution and is well accepted in existing literature and common discourse.

### Statistical analysis

#### Bivariate copula regression model

In this study, we jointly model childhood stunting and wasting while accounting for the spatial distribution of their association across Myanmar. For that purpose, we use a bivariate copula regression model that incorporates spatial effects at the regional level as well as flexible non-linear functions of covariates. The resulting modelling framework is known as a copula geoadditive model^(25)^. In this framework, the dependence structure between the responses is modelled using copulas, which are functions that enable flexible specification of the marginal models of the responses separately from that of the joint distribution governing their dependence structure^(28)^. Although a relatively new concept, copulas have been extensively used for modelling association between diverse classes of responses across multiple fields. Applications range from modelling mixed binary-continuous data^(29)^, continuous and discrete longitudinal data^(30)^, censored data^(31)^ and count data^(32)^. Copula models have been used in finance and insurance^(33,34)^, forestry and environment^(35)^ and marketing^(36)^ as well. Excellent reviews of copula models are provided by Trivedi and Zimmer^(37)^ and Genest^(38)^.

Assuming 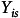 and 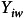 to be the stunting and wasting status of the 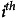 child such that 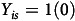 if the child is stunted (not stunted) and 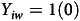 if the child is wasted (not wasted), the copula structure enables specification of the joint probability of the 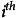 child being stunted as well as wasted, that is, 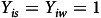 as

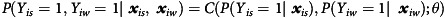

where 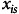 and 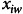 are the vector of determinants of stunting and wasting, respectively. Here, 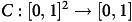 is a two-place copula function, while 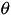 , known as the copula parameter, quantifies the dependence between stunting and wasting prevalence among the children^(26)^. A latent variable representation of binary probabilities is used to parameterize 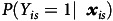 as

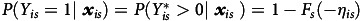

where 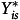 is a continuous latent variable expressible as 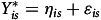 , 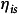 being the linear predictor of consisting of linear, non-linear and spatial effects while 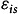 is a white noise error. 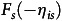 is the cumulative distribution function of the error and determines the structure of the marginal model linking the stunting indicator 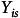 to the corresponding linear predictor. The flexibility of the copula approach lies in the fact that 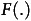 can correspond to a broad class of univariate distributions (Gaussian, logistic, Gumbel for instance) depending on the assumed distributional form for the error term. For instance, a standard normal distributional assumption for 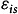 would lead to a probit specification for the corresponding marginal model. A similar setup can be replicated for the wasting indicator, 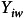 .

#### Marginal model specification

We incorporate four types of effects in the marginal models for stunting and wasting, namely (i) regular fixed effects of the categorical variables and of those continuous variables, which are linearly related to the response; (ii) flexible non-linear effects for variables, which have a curvilinear association with the response; (iii) within-region (unstructured) spatial effects to account for the presumed similarity in stunting and wasting prevalence among children residing in the same region and lastly (iv) between-region (structured) spatial effects to account for the assumed dependence in stunting and wasting prevalence among children residing in adjacent regions. The non-linear effects are estimated by thin-plate regression splines while the structured spatial effects are estimated by Markov random field smoother, which is based on the neighbourhood structure of the regions^(26,29)^.

#### Model selection

The flexibility of the copula approach enables the selection of the optimal copula representation for modelling the dependence between the responses independently of the structure of the marginal models. Accordingly, we employed a two-step approach for selection of the optimal framework, namely (i) assuming a probit representation for each of the marginal models, we selected the copula representation that produced the lowest values of Akaike information criterion (AIC) and Bayesian information criterion (BIC) across multiple copula choices; (ii) given the optimal copula representation so obtained, we selected the marginal model structure that corresponded to the lowest AIC and BIC values across competing marginal models. Accordingly, we chose the survival Gumbel copula along with a complementary log–log link for the marginal model of wasting and probit link for the marginal model of stunting since it corresponded to the lowest values of AIC as well as BIC. The AIC and BIC values for all the competing models are provided in the supplementary document.

In addition to the marginal models of stunting and wasting, we modelled the copula parameter, 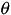 , with respect to the regions in order to capture any spatial variation in the association between stunting and wasting. This may enable the identification of regions where stunting and wasting are strongly or weakly associated, which, in turn, can provide valuable insights to policy makers on the need for region-specific interventions. For ease of interpretation, the copula parameter was transformed to Kendall’s tau correlation coefficient ( 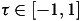 ), which is a measure of the degree of concordance between two variables^(26)^. Accordingly, variation in the region-specific 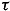 values would be indicative of a spatial variation of the dependence between stunting and wasting across Myanmar.

Analysis was carried out using the R package *GJRM* (generalised joint regression modelling)^(26,39)^ while mapping was carried out in QGIS 3.22 using shapefiles freely obtainable from the Spatial Data Repository maintained by the DHS Program (https://spatialdata.dhsprogram.com/boundaries). All estimates have been weighted using the sampling weights provided in the DHS data file. The standard errors of all the estimates have been suitably adjusted to account for the multistage cluster sampling carried out in the MDHS. Statistical significance has been assessed at the customary 5 % significance level except for sparse data situations in which the more liberal 10 % level has been used.

## Results

### Sample characteristics

Table 1 shows the weighted prevalence and corresponding 95 % confidence intervals of stunting, wasting and both corresponding to the categorical attributes considered in the study. Overall, the weighted prevalence of stunting, wasting and both stunting and wasting was 29 %, 5 % and 1·7 %, respectively. No tangible differences in the prevalence of wasting or both stunting and wasting were seen across the categories of any of the child, maternal and household attributes. However, stunting prevalence did reveal interesting features across the various attributes. For instance, males had higher stunting prevalence than females, a pattern that was true for wasting and stunting and wasting as well. Children belonging to ethnic minority regions had considerably higher stunting prevalence (33 %) than those belonging to ethnic majority regions (24 %). Similarly, children of working mothers had higher stunting prevalence (31 %) than those belonging to non-working mothers (20 %). There was a steady decline in stunting prevalence with increase in maternal educational attainment and household wealth. Children hailing from rural areas had a much higher stunting prevalence (31 %) than those belonging to urban locations (20 %). Gender of household head did not seem to have any effect on the prevalence of stunting, wasting or both. However, lower stunting prevalence was observed in households having access to improved toilet facility and in-house drinking water compared to households having restricted or no access to such facilities. Water treatment did not seem to have any effect on stunting prevalence.

**Table 1:** Marginal and concurrent prevalence of stunting, wasting and both stunting and wasting for children under 5 in Myanmar

		Stunting		Wasting		Stunting and wasting	
	Sample size	Wt %	95 % CI	Wt %	95 % CI	Wt %	95 % CI
Child characteristics							
Gender							
Male	2150	0·30	0·28, 0·32	0·05	0·04, 0·06	0·018	0·01, 0·02
Female	2012	0·27	0·25, 0·29	0·04	0·03, 0·05	0·015	0·01, 0·02
Ethnicity							
Majority	2063	0·24	0·22, 0·26	0·06	0·05, 0·07	0·02	0·01, 0·03
Minority	2099	0·33	0·31, 0·35	0·04	0·03, 0·05	0·016	0·01, 0·02
Maternal characteristics							
Working status							
Working	2163	0·31	0·29, 0·33	0·04	0·03, 0·05	0·017	0·01, 0·02
Not working	1999	0·26	0·24, 0·28	0·06	0·05, 0·07	0·017	0·01, 0·02
Education							
No	695	0·37	0·33, 0·41	0·04	0·02, 0·05	0·03	0·01, 0·04
Primary	1880	0·31	0·29, 0·33	0·05	0·04, 0·06	0·02	0·01, 0·03
Secondary	1295	0·24	0·22, 0·26	0·05	0·04, 0·06	0·01	0·005, 0·015
Higher secondary	295	0·17	0·13, 0·21	0·08	0·05, 0·11	0·00	0·00, 0·00
Household characteristics							
Residence							
Rural	3308	0·31	0·29, 0·33	0·04	0·03, 0·05	0·017	0·01, 0·02
Urban	854	0·20	0·17, 0·23	0·07	0·05, 0·09	0·015	0·01, 0·02
Household head							
Male	3490	0·29	0·27, 0·30	0·05	0·04, 0·06	0·017	0·01, 0·02
Female	672	0·28	0·25, 0·31	0·04	0·02, 0·05	0·015	0·01, 0·02
Wealth							
Richest	492	0·15	0·12, 0·18	0·08	0·06, 0·10	0·01	0·00, 0·02
Richer	700	0·21	0·18, 0·24	0·04	0·02, 0·05	0·01	0·00, 0·02
Middle	769	0·29	0·26, 0·32	0·05	0·03, 0·06	0·02	0·01, 0·03
Poorer	947	0·32	0·29, 0·35	0·04	0·03, 0·05	0·014	0·01, 0·02
Poorest	1254	0·36	0·33, 0·39	0·05	0·04, 0·06	0·03	0·02, 0·04
Toilet facility							
Improved	2202	0·26	0·24, 0·28	0·05	0·03, 0·07	0·014	0·01, 0·02
Unimproved	1960	0·31	0·29, 0·33	0·05	0·04, 0·06	0·02	0·01, 0·03
On-premise drinking water							
Available	3167	0·27	0·25, 0·28	0·05	0·04, 0·06	0·016	0·01, 0·02
Not available	995	0·34	0·31, 0·37	0·05	0·04, 0·06	0·02	0·01, 0·03
Treat water							
Yes	3100	0·29	0·27, 0·31	0·05	0·05, 0·06	0·016	0·01, 0·02
No	1062	0·29	0·26, 0·32	0·06	0·05, 0·07	0·02	0·01, 0·03
Overall	4162	0·29	0·28, 0·30	0·05	0·04, 0·06	0·017	0·01, 0·02

Wt%: weighted prevalence with weights being the sampling weights.

**Fig. 1:**
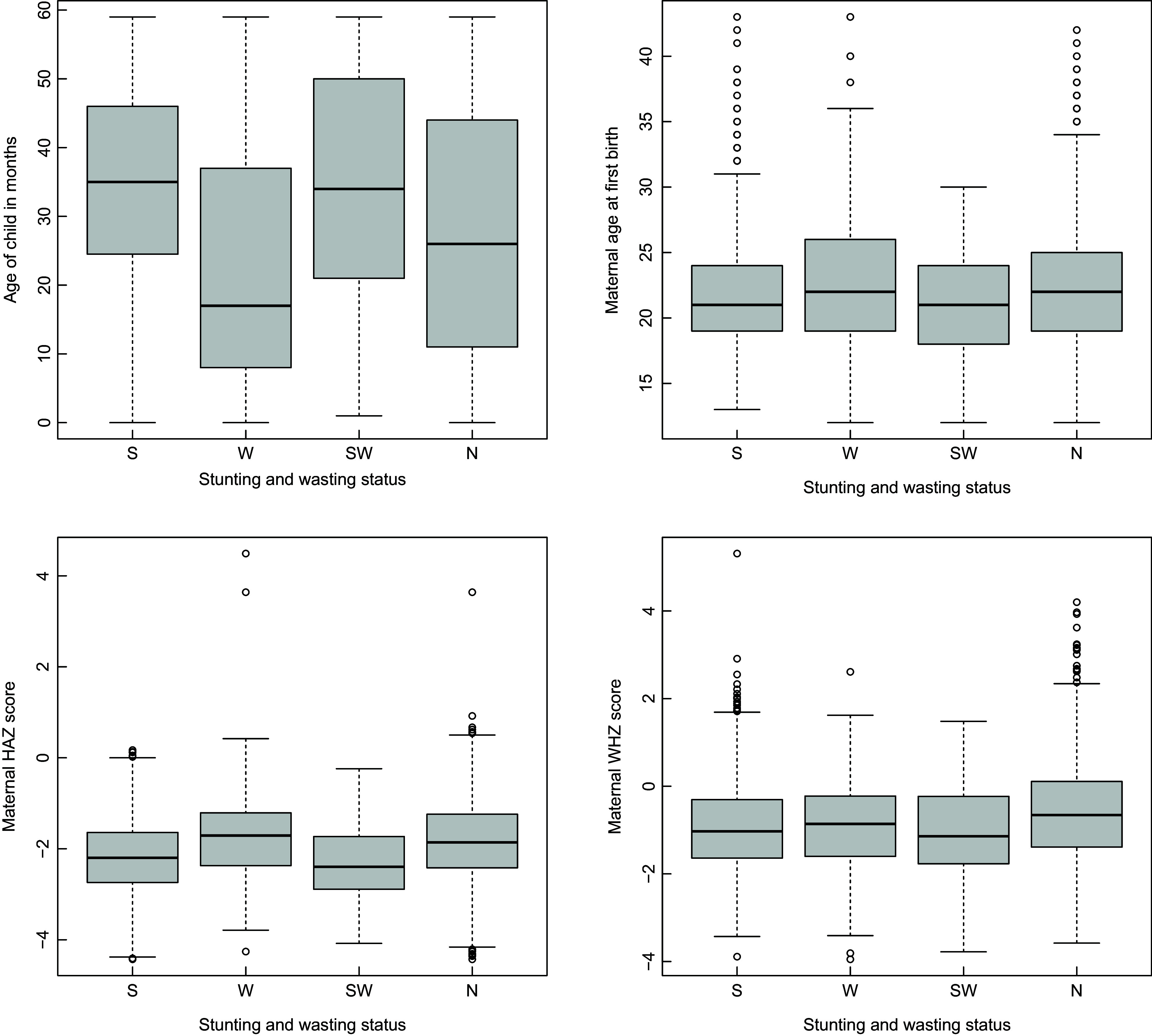
Boxplots of continuous variables by stunting and wasting status of child. Here ‘S’, ‘W’, ‘SW’ and ‘N’ imply ‘only stunted’, ‘only wasted’, ‘stunted as well as wasted’ and ‘not stunted or wasted’, respectively

Figure 1 depict the boxplots of the continuous covariates, namely age of child (in months), maternal HAZ and WHZ scores and maternal age at first birth. For each of the covariates, separate box plots are constructed for children who are stunted, wasted, both stunted and wasted and who are neither stunted nor wasted. Each of the box plots reveals the minimum, maximum as well as first, second and third quartiles of the respective covariates for each of the child samples.

Wasted children seem to have the lowest median age among the four groups, which was much lower than that of the other three groups. In fact, the median ages of stunted and stunted and wasted children were comparable to the third quartile of the ages of wasted children. Maternal age of first birth corresponding to wasted children was higher, on average than that of the other three groups. The median standardised HAZ scores of mothers who had stunted as well as stunted and wasted children were lower than that of mothers who had wasted or healthy children. However, no tangible difference in the distribution of the maternal WHZ scores were observed across the four samples.

Table 2 cross classifies the observed sample of 4162 children according to their stunting and wasting status. Overall, stunting and wasting prevalence were 30·39 % and 6·65 %, respectively, while 1·68 % children were both stunted and wasted. A Pearsonian Chi-square test of association generated a test statistic with a *P*-value of 0·064, which was significant at 10 % significance level, thus implying mild association between stunting and wasting.

**Table 2:** 2 × 2 contingency table cross-classifying the sampled children according to their stunting and wasting status

	Stunting status		
Wasting status	Stunted	Non-stunted	Total
Wasted	70	207	277
Non-wasted	1195	2690	3885
Total	1265	2897	4162

### Bivariate copula model

As part of the bivariate copula model, three distinct models are fitted, namely marginal model for stunting, marginal model for wasting and the model for the copula parameter. Each of the marginal models incorporates fixed effects of the categorical covariates, non-linear effects of the continuous covariates as well as separate unstructured and structured spatial effects to accommodate for within and between-region correlations in stunting and wasting, respectively. The model for the copula parameter accounts for spatial variation in the association between stunting and wasting across the regions of Myanmar. All the categorical and continuous covariates were accommodated in both the marginal models. Various modelling frameworks were considered with varying copula specifications and marginal model structures and the one with the lowest AIC was adjugated as the optimal one. For this study, the optimal model corresponded to a complementary log–log link for the marginal model for wasting, a probit link for the marginal model for stunting and a survival Gumbel copula function for the dependence between the two responses.

### Fixed effect results

Table 3 shows the parameter estimates, standard errors and *P* values corresponding to the categorical predictors included in the marginal models for stunting and wasting. Based on the results, boys and children belonging to ethnic minority regions are significantly more likely to be stunted compared to females and ethnic minority children (gender estimate = 0·138, *P* value = 0·007; ethnicity estimate = –0·20, *P* value = 0·0003). The likelihood of stunting progressively decreased with increasing household wealth. Specifically, children belonging to households in the two highest wealth quantiles are significantly less likely to be stunted than those belonging to the poorest households (quintile 5 wealth estimate = –0·28, *P* value = 0·02; quintile 4 wealth estimate = –0·20, *P* value = 0·04). Maternal working status, maternal educational attainment, household location (urban or rural), drinking water or sanitation and gender of household head did not seem to have any significant association with stunting prevalence controlling for the other factors.

**Table 3:** Parameter estimates, standard errors and *P* values for the fixed effects for the bivariate copula regression model for stunting and wasting

	Stunting			Wasting		
	Estimate	Standard error	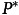	Estimate	Standard error	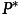
Child characteristics						
Gender						
Male	0·138	0·051	0·007	0·229	0·134	0·09
Ethnicity						
Majority	−0·198	0·055	0·0003	0·193	0·159	0·22
Maternal characteristics						
Working status						
Working	0·089	0·061	0·15	−0·223	0·146	0·13
Education						
Primary	−0·029	0·076	0·70	−0·037	0·212	0·86
Secondary	−0·110	0·097	0·26	−0·05	0·257	0·85
Higher secondary	−0·054	0·157	0·73	−0·028	0·323	0·93
Household characteristics						
Residence						
Rural	0·056	0·068	0·41	−0·327	0·202	0·09
Household head						
Male	−0·043	0·083	0·60	0·218	0·201	0·28
Wealth						
Poorer	−0·011	0·079	0·88	−0·268	0·205	0·19
Middle	−0·042	0·090	0·64	0·042	0·237	0·86
Richer	−0·196	0·096	0·04	−0·487	0·271	0·07
Richest	−0·276	0·123	0·02	−0·165	0·287	0·56
Toilet facility						
Improved	−0·063	0·061	0·30	0·084	0·161	0·60
On-premise drinking water						
Available	−0·075	0·071	0·29	−0·221	0·192	0·25
Treat water						
Yes	0·008	0·073	0·91	−0·086	0·173	0·62

*Statistical significance was assessed at 5 % level of significance unless stated otherwise.

Childhood wasting had significant association with household wealth as well as with child gender and household location, albeit at 10 % significance level. Specifically, children hailing from richer households were significantly less likely to be wasted than those from the poorest households (quintile 4 wealth estimate = –0·49, *P* value = 0·07). Children hailing from rural regions had lower prevalence of wasting than those hailing from urban regions (region estimate = –0·33, *P* value = 0·09). Lastly, boys had significantly higher likelihood of wasting (gender estimate = 0·23, *P* value = 0·09). Remaining variables such as childhood ethnicity, access to proper sanitation or drinking water, gender of household head or maternal educational attainment did not have any significant association with the prevalence of wasting.

### Non-linear and spatial effects

Table 4 depicts statistical significance for the non-linear and spatial terms for both stunting and wasting. Age of child and maternal WHZ score have significant non-linear effects on the likelihood of stunting as well as wasting at 1 % significance level while maternal HAZ score and age at first birth have significant non-linear effect on childhood stunting at the 1 % and 5 % levels, respectively. However, none of these two variables seems to have any significant association with childhood wasting.

**Table 4:** Chi-square test statistic and associated *P*-values for the non-linear and spatial components of the bivariate copula model for stunting and wasting

	Stunting		Wasting	
	Chi-square value	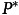	Chi-square value	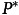
Age of child (months)	180·31	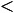 0·001	32·02	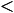 0·001
HAZ score of mother	76·09	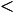 0·001	4·03	0·18
WHZ score of mother	28·30	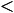 0·001	29·72	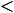 0·001
Maternal age at first birth	8·74	0·047	10·23	0·18
Unstructured spatial effect	5·84	0·036	15·23	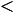 0·001
Structured spatial effect	1·46	0·069	2·86	0·003

HAZ, height-for-age Z score; WHZ, weight-for-height Z score.

*Statistical significance was assessed at 5 % level of significance.

**Fig. 2:**
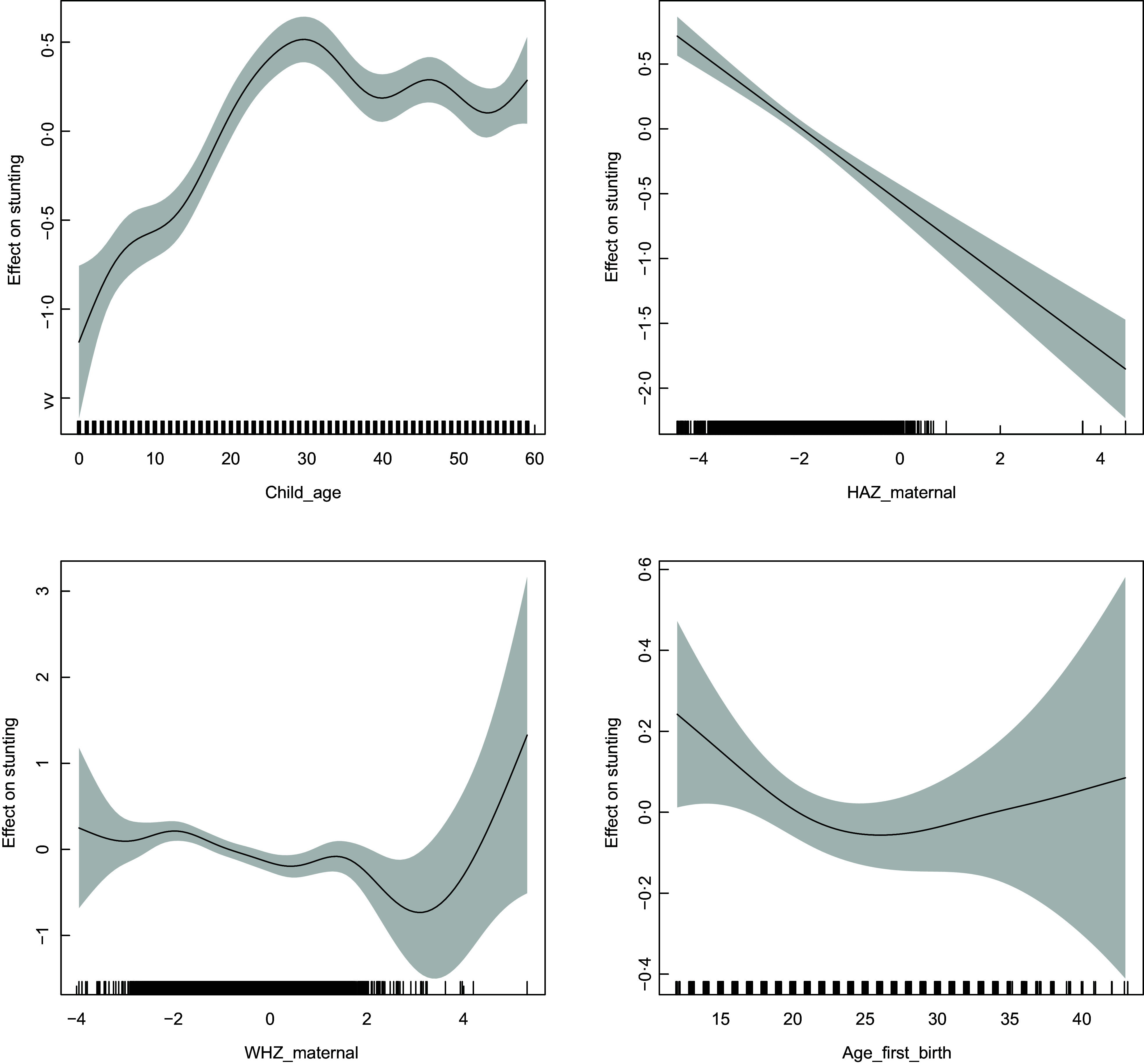
Estimated non-linear effects of child’s age, maternal HAZ score, maternal WHZ scores and maternal age at first birth on the likelihood of stunting. HAZ, height-for-age Z score; WHZ, weight-for-height Z score.

**Fig. 3:**
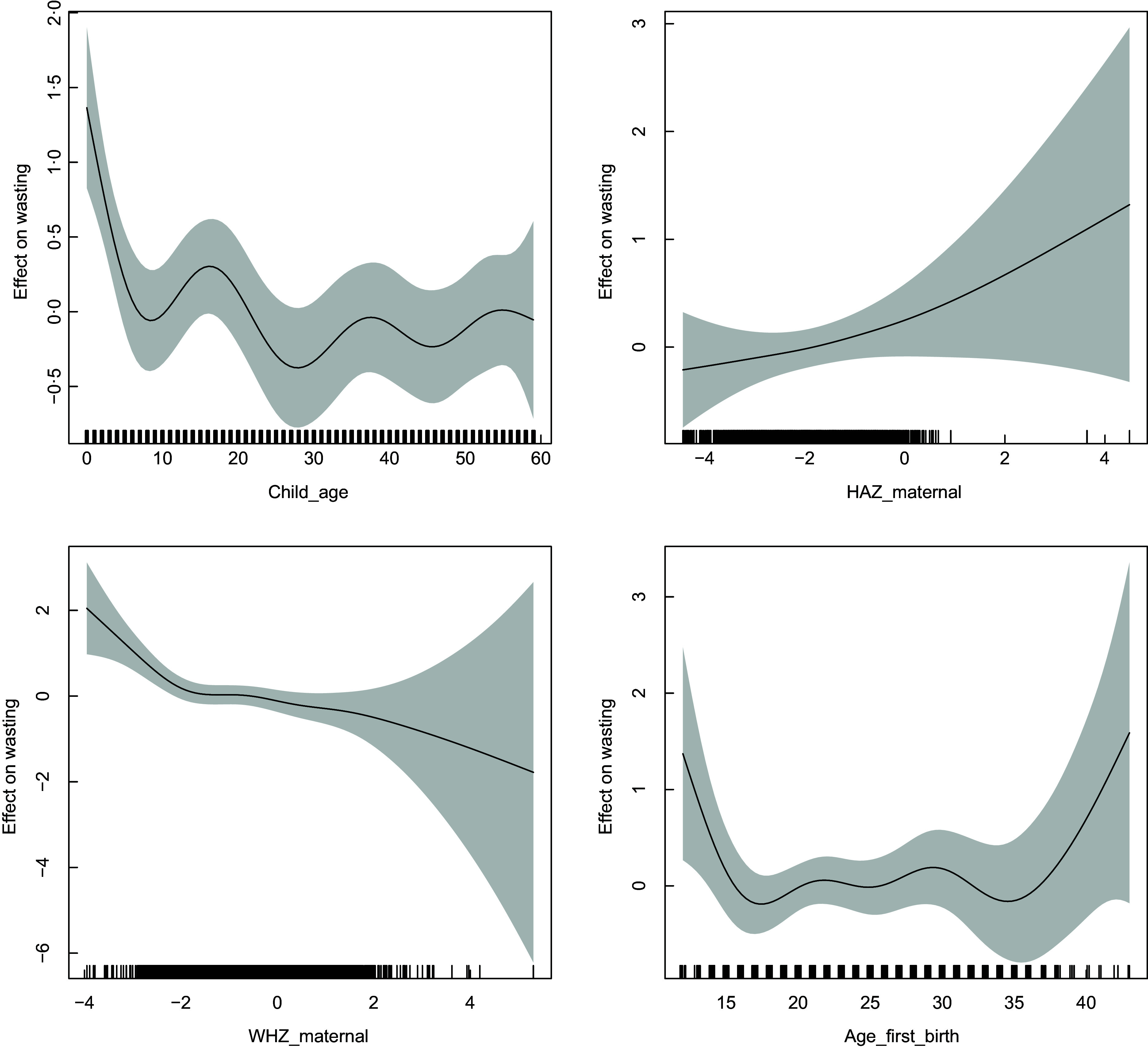
Estimated non-linear effects of child’s age, maternal HAZ score, maternal WHZ scores and maternal age at first birth on the likelihood of wasting. HAZ, height-for-age Z score; WHZ, weight-for-height Z score.

Figures 2 and 3 depict the non-linear patterns of these variables on the likelihood of stunting and wasting. The likelihood of stunting steadily increases with child’s age till about 30 months post which it declines. However, wasting seems to have an overall decreasing trend with age with noticeable fluctuations. Moreover, the prevalence of wasting declines steadily with an increase in maternal WHZ scores implying that wasted mothers have a considerably higher likelihood of having wasted children and vice versa. Finally, the likelihood of stunting reduces with higher age at first birth until about 25 years, post which it gradually increases. On the other hand, likelihood of wasting has a sharp drop between 12 years till 18 years and remains fairly stable till about 35 years of age post which there is again a sharp increase.

**Fig. 4:**
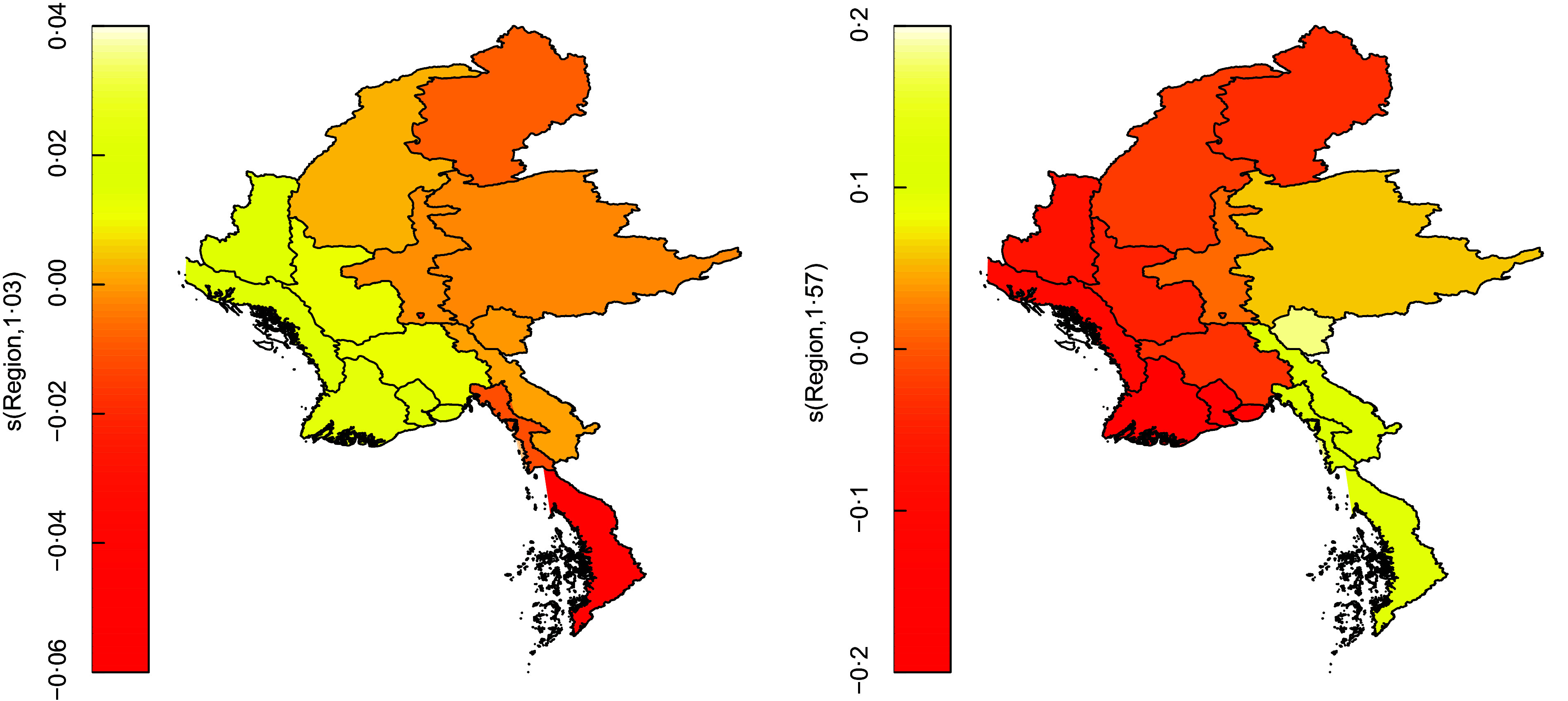
Structured spatial variation of stunting and wasting

The unstructured spatial effects of both stunting and wasting are significant at 5 % level implying that stunting and wasting prevalence have strong within-region variations. Moreover, the structured spatial effect of wasting is significant at 5 % level while that of stunting is significant at 10 % level. This implies that childhood wasting has strong between-region variation in Myanmar while that of stunting is mild. Figure 4 provides a visual depiction of the structured spatial effects of stunting and wasting, respectively. In both the figures, darker or reddish zones correspond to regions of lower incidence while lighter or yellowish zones correspond to regions of higher incidence of stunting and wasting. It is clear that wasting has a higher degree of variation across the country with eastern and southern regions like Kayah, Kayin, Mon, Tanintharyi and Shan recording the highest prevalence while western and south-western regions like Rakhine, Ayeyarwaddy and Yangon having the lowest prevalence. However, stunting has the opposite pattern with higher prevalence in western states like Chin, Rakhine, Ayeyarwaddy, Yangon, Magway and Bago and lower prevalence in the southern states like Mon and Tanintharyi. Two critical insights can be derived from these maps. First, stunting and wasting have mild association, which is indicative of the presence of other factors that drive their prevalence. Second, any socio-economic or nutritional intervention should account for these regional variations for impact maximisation.

### Association between stunting and wasting

**Fig. 5:**
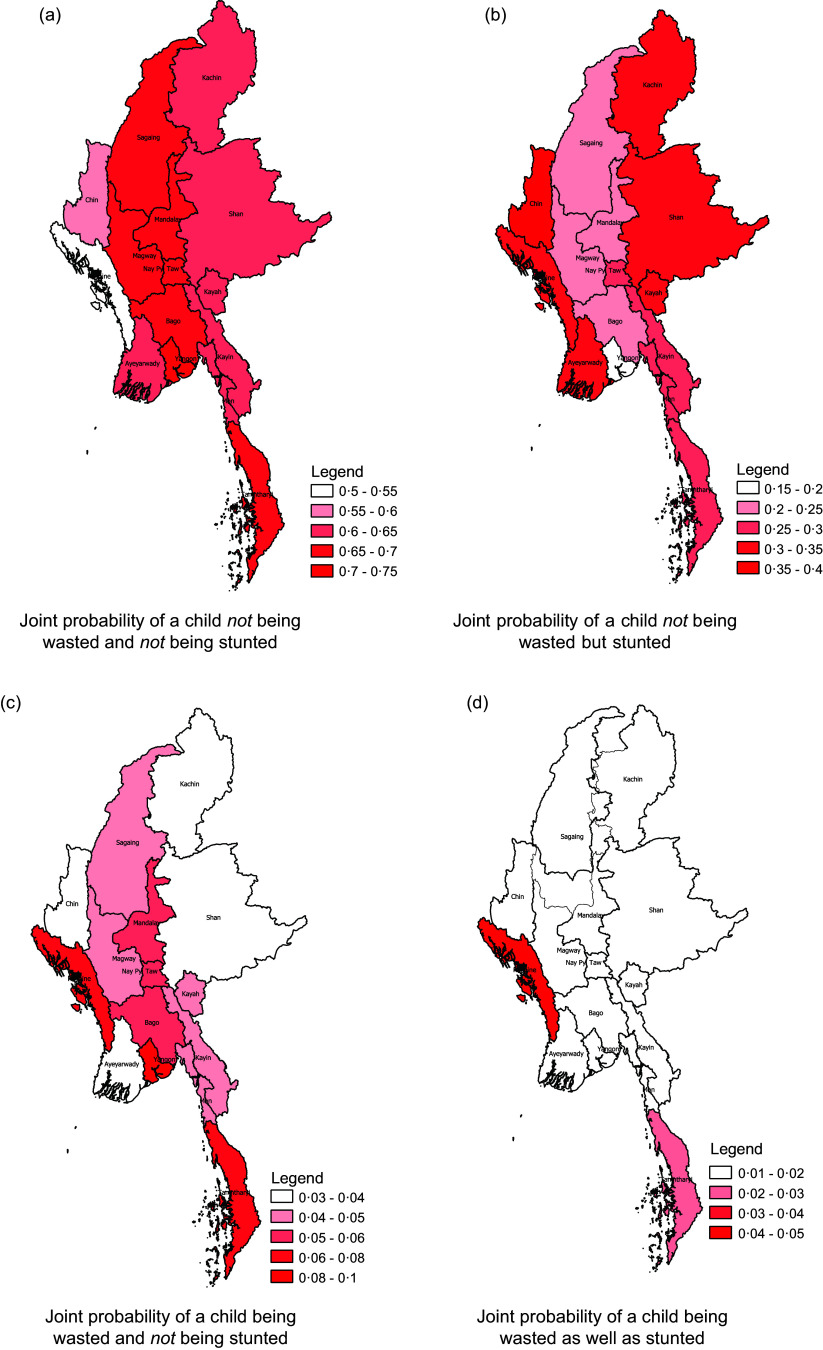
Joint probability maps of stunting and wasting

The copula parameter 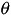 was set to vary across regions through a Markov random field specification. The estimated value of the parameter, averaged over the 15 regions, was 1 with a 95 % CI^(1,17)^. The corresponding Kendall’s tau coefficient was close to zero with a 95 % CI of (0, 0·941). The values remained similar across the regions implying mild, homogeneous positive association between stunting and wasting. We averaged the estimated joint probabilities of stunting and wasting for each of the fifteen regions based on the bivariate copula model while accounting for this inherent association. Specifically, the joint probabilities correspond to four distinct events of a child being neither stunted or wasted, being stunted but not wasted, being wasted but not stunted and being stunted as well as wasted. The spatial maps of the joint probabilities are shown in Fig. 5.

As per Fig. 5(a), the joint probabilities of a child not being stunted and wasted are quite high across Myanmar. This is also corroborated from Fig. 5(d), which depicts low joint probabilities of a child being both stunted and wasted throughout the country. Fig. 5(b) indicates that coastal regions to the west and those in the east bordering China have higher joint probabilities of a child being stunted but not wasted compared with that in the central and southern regions. Similarly, Fig. 5(c) indicates that the joint probability of a child being wasted but not stunted is quite low across the country.

## Discussion

This study is devoted to exploring the spatial variation and socio-economic drivers of childhood stunting and wasting in Myanmar while accounting for any inherent dependence between the two. This is accomplished using a bivariate copula regression model, which enables us to incorporate linear and non-linear covariate effects as well as within- and between-region spatial variation in the responses. The copula framework enables the separation of the dependency structure of the responses from their marginal models and hence the estimation routines^(26)^. The flexibility of the copula approach over typical multivariate analysis is that it does not require the normality and linearity assumptions for modelling the dependence between responses^(28)^. Moreover, it can seamlessly incorporate various types and associated combination of responses like binary, ordinal, count and continuous in both cross-sectional and longitudinal settings^(25,29–32)^. Specifically for the current study, the copula framework enables us to produce spatial maps of the joint probabilities of stunting and wasting prevalence as well as quantify their association across the regions of Myanmar. These maps can provide crucial data-driven insights about regional variations in the joint prevalence of childhood stunting and wasting, which, in turn, can aid in the formulation of region-specific interventions to arrest and reverse the progression of these ailments. To the best of our knowledge, this is the first study that deploys such a flexible framework for joint modelling of stunting and wasting among children under 5 on nationally representative survey data of any country in general and Myanmar in particular.

Overall, the prevalence of stunting and wasting in the country were 29 % and 5 %, respectively, while that of both stunting and wasting prevalence was only 1·7 %. The marginal prevalence of wasting and concurrent prevalence of both stunting and wasting was uniformly low across all categories of the sampled children. However, the marginal occurrence of stunting and concurrent occurrence of both stunting and wasting was higher among children belonging to stunted mothers compared to those belonging to healthy mothers.

The copula modelling results indicate mild positive association between childhood stunting and wasting that remains homogeneous throughout the country. However, marked differences were observed in the spatial distribution of stunting and wasting prevalence across the regions. Specifically, eastern regions bordering the Indian Ocean have higher prevalence of stunting but lower prevalence of wasting while southern regions have higher prevalence of wasting but lower prevalence of stunting. The overall best performing regions were Mandalay and Naypyidaw, the capital. This indicates that in terms of policy formulation and implementation, a ‘one-size-fits-all’ strategy may be sub-optimal and calls for a more nuanced, region-specific intervention framework. Spatial maps of the joint probabilities of stunting and wasting depicted low likelihood of their dual occurrence throughout the country, thus corroborating the positive strides taken by Myanmar in recent years towards achieving the global nutritional targets^(40)^. However, there were noticeable variations in the joint likelihood of stunting but no wasting across the country. Specifically, coastal regions in the east and mountainous regions in the northwest had higher estimated joint probabilities of a child being stunted but not wasted compared to the central and southern part of the country. While corroborating that stunting and wasting are associated, albeit mildly, these maps are also indicative of the presence of additional drivers for stunting other than wasting, specifically in the eastern and western regions. Hence, effective policy formulation should account for regional attributes for impact maximisation.

Analysis of the marginal models of stunting and wasting yielded expected patterns of association between socio-economic and demographic variables and the likelihood of stunting and wasting. Specifically, boys and children hailing from poorer households had significantly higher susceptibility to stunting as well as wasting, which is consistent with those available in the existing literature on childhood undernutrition for low- and middle-income countries^(41–43)^. Maternal working status had mild association with stunting and wasting prevalence but in opposite directions. Specifically, children belonging to working mothers had a lower risk of being wasted but a higher risk of being stunted. Moreover, children from rural areas had a significantly lower risk of being wasted compared with their urban counterparts. Interestingly, ethnicity had significant association with stunting prevalence with ethnic minority children being at a significantly higher risk of being stunted compared with their majority counterparts. This was a relevant finding since the ethnic minorities of Myanmar have been subjected to severe persecution and discrimination by the military which has ruled the country since its independence. Similar findings on ethnic disparities in childhood undernutrition have been reported in studies from Vietnam and Latin America^(44–46)^. Maternal educational attainment, gender of household head and sanitation and drinking water facilities did not have any noticeable effect on the likelihood of stunting and wasting.

In addition to the fixed effects, children’s age, maternal HAZ and WHZ scores as well as maternal age at first birth had complex non-linear association with the likelihood of stunting and wasting. Specifically, stunted and wasted mothers are more likely to have stunted children while the same is true for overweight mothers as well. The results are also indicative of the fact that having the first child post 30 years increases the chance of stunting while conceiving before 18 years or after 35 years increases the odds of having a wasted child. Overall, the results are indicative of the necessity of deploying flexible modelling frameworks to decipher the complex association patterns between various maternal attributes and the prevalence of stunting and wasting. These findings add to the insights available in the existing literature on childhood undernutrition in Myanmar^(11,14,24)^.

This study is not free from limitations, which may provide pointers for future research. The limitations are model-based as well as data-based. As far as the former is concerned, the copula framework, despite its varied advantages and flexibility, has limitations in terms of non-uniqueness of copulas for modelling discrete and mixed outcomes, which leads to difficulty in the interpretation of results. Second, the usage of Markov random field smoother for estimating structured spatial effects relies on the assumption of correlated responses across adjacent areas, a premise that is often difficult to verify. Third, the specification of a particular copula heavily relies on the validity of assumption about the nature of dependencies of the responses, both Gaussian and non-Gaussian. Lastly, a major challenge for copula models lies in implementing a robust model selection and validation mechanism that simultaneously incorporates selection of optimal marginal models, predictor specification and conditional dependence structure between the responses. This is made all the more difficult due to the non-generalisability of standard tools of model checking (say, quantile–quantile plots) for correlated responses, specially binary ones.

As far as data-based limitations are concerned, first and foremost, the Myanmar Demographic and Health Survey data have only been collected once, in 2015–2016. The cross-sectional nature of these data limited the scope for performing causal inference. Second, environmental predictors such as cluster height, vegetation index and land surface temperature have not been accounted for in the modelling framework, which, if done, can further our understanding about the relationship between environmental drivers and the likelihood of stunting and wasting. Third, socio-economic and demographic variables can be incorporated in the model for the copula parameter in addition to the region-level structured spatial effect. Doing so can yield further insights about the dependence between stunting and wasting and their association with maternal and household-specific variables.

The main contribution of this study is threefold. First, precise spatial mapping of the marginal prevalence of stunting and wasting across regions of Myanmar accounting for both within and between-region variation. Second, spatial delineation of the various combinations of the joint prevalence of stunting and wasting. Third, deciphering the underlying non-linear association between childhood age and maternal HAZ and WHZ scores, thus enabling a more nuanced understanding of the effect of maternal stunting and wasting status on that of the children’s.

The findings of our study can be utilised for crafting targeted policies and programmes towards improving the state of childhood undernutrition in Myanmar. An essential component of successful implementation of any such policies is proper identification of the most vulnerable subgroups and regions of the country. The fixed and spatial effects obtained from the study provide necessary pointers towards achieving that goal. What stands out in that process is the necessity of accounting for the implicit dependence between various indices of childhood undernutrition for better understanding of the mechanisms through which those can be tackled through effective, region-specific policy formulations. This study provides an alternative and more robust framework for achieving the same compared with typical multivariate models that fail to account for such subtleties.
